# SR-FTIR Biomolecular Characterization of the Hippocampus: The Role of Tenascin C in Adult Murine Neurogenesis in the Subgranular Zone

**DOI:** 10.3390/cells14060435

**Published:** 2025-03-14

**Authors:** Milena Korenić, Andrej Korenić, Vera Stamenković, Tanja Dučić, Pavle Andjus

**Affiliations:** 1 Institute of Physiology and Biochemistry “Jean Giaja”, Faculty of Biology, University of Belgrade, 11000 Belgrade, Serbia; milena.tucic@bio.bg.ac.rs (M.K.); andrej.korenic@bio.bg.ac.rs (A.K.); 2 Center for Integrative Brain Research, Seattle Children’s Research Institute, Seattle, WA 98105, USA; vera.stamenkovic@seattlechildrens.org; 3 ALBA-CELLS Synchrotron, 08290 Cerdanyola del Vallès, Spain; tducic@cells.es

**Keywords:** hippocampal layers, subgranular zone, SR-FTIR, tenascin C

## Abstract

To better understand adult neurogenesis, the biomolecular specificity of the subgranular zone should be investigated in comparison to other layers of the hippocampus. Adult neurogenesis occurs at a reduced rate in adulthood compared to the period of development, but it can be increased with exposure to an enriched environment (EE). This can be used to investigate the regulatory role of molecules present in the extracellular matrix, such as tenascin C (TnC). This study, using Synchrotron radiation Fourier Transform Infrared spectroscopy (SR-FTIR), shows that the differences between the hippocampal layers in adolescence are maintained as subtle and significant in adulthood. The main difference in FTIR spectra was observed for nucleic acid and carbohydrate and for the comparison of the subgranular zone (SGZ) with hippocampal CA3. Moreover, we have detected changes in the protein and nucleic acid content of the SGZ that accompany the process of neurogenesis under the influence of an enriched environment. The latter effects are, however, lacking in mice with a gene ablation for tenascin C. Overall, these results show that observed discrete biomolecular differences in hippocampal layers follow the rate of neurogenesis that is enhanced by EE and dependent on TnC.

## 1. Introduction

The nervous system possesses the capacity to respond to experience or injury via structural or functional modification—a phenomenon known as neuronal plasticity—which involves changes in gene transcription, cell composition, morphology and excitability [1]. For example, the hippocampus shows a specific form of neuronal plasticity since the modification of dendritic complexity, synapse number and formation of entirely novel neuronal connections occurs not only during development but also in adulthood during neurogenesis [2,3]. The principal neurons, the pyramidal neurons, in the hippocampus are organized in successive layers named *Cornu Ammonis* (CA) 1, 2 and 3, with granular neurons forming the dentate gyrus (DG)—arrow-like structure in impinging on the CA3 layer. These neurons have distinctive cell morphologies, neurogenic potential, synaptic connections, signaling pathways and resistance to damaging conditions [4,5].

The biomolecular composition of the cell—including macromolecules such as lipids, proteins, nucleic acids and carbohydrates—is the basis and product of the biochemical processes that take place within it. Appositely, FTIR spectroscopy measures the absorption of IR light by chemical bonds and provides information about the content, structure and conformation of macromolecules [6]. Furthermore, coupling FTIR spectroscopy with a synchrotron (SR) infrared beam is suitable for studying biological samples since it is non-destructive, label-free and of improved resolution as compared with other techniques such as conventional FTIR or Raman spectroscopy [6,7]. These advantages allow for the acquisition of information from an intact sample without altering biological structures [8]. Therefore, SR-FTIR spectroscopy is applicable for the biomolecular characterization of layers in the hippocampus tissue. Thus, Dudala and colleagues used SR-FTIR to investigate the distribution of proteins and lipids in the molecular, multiform and granular layer of the rat hippocampus [9] while a more detailed study used FTIR imaging to monitor the molecular changes in the rat hippocampus after traumatic brain injury and to investigate the protein/lipid dynamics, the metabolic ratio and the oxidative stress parameters [10].

Adult neurogenesis is a distinctive feature of the adult hippocampus that allows for continuous adaptation of hippocampal circuitry accompanying the generation of new neurons [11]. The extracellular matrix molecules in the SGZ have an important role in adult neurogenesis and one of the specifically enriched molecules is tenascin C (TnC) [12]. TnC is a regulatory glycoprotein that is highly expressed in the extracellular matrix (ECM) during development and tissue remodeling. It interacts with many ECM molecules, such as proteoglycans and fibronectin, and cell receptors, such as integrins and Toll-like receptor 4 (reviewed in [13,14]). The role of TnC in the regulation of adult neurogenesis in the hippocampus is still being investigated, but there are a number of studies that confirm its role in the processes related to adult neurogenesis, such as proliferation, cell adhesion and migration (reviewed in [15,16]). It was shown that TnC regulates the mitogenic response of oligodendrocyte progenitors, depending on the signaling of integrins and platelet-derived growth factor [17,18]. Also, radial glial cells (a subtype of stem cells in the embryonic CNS) grown in neutrospheres from murine CNS show a reduced number in the absence of TnC [19]. Furthermore, adult hippocampal neurogenesis is a process highly sensitive to experience [20], such as the one induced by exposure to an enriched environment (EE), a protocol known to induce brain structural and functional plasticity [21,22]. Thus, EE can contribute to the survival of new granule neurons in the rat SGZ [23]. Also, physical activity, such as running, increases cell proliferation and survival of newborn neurons in the rat SGZ [24]. In clinical studies, adult neurogenesis has been associated with the pathogenesis and treatment of neuropsychiatric disorders in clinical settings. For instance, diminished neurogenesis has been correlated with cognitive deficits and atypical dendritic branching in individuals with schizophrenia [25]. Furthermore, rehabilitative strategies such as physical exercise and environmental enrichment have proven effective in enhancing adult neurogenesis in animal studies and have the potential to alleviate symptoms of depression and cognitive decline [26].

We hypothesized that genetic modifications, in interaction with environmental factors, shape the composition of the adult neurogenic niche. Although specific properties of the microenvironment where adult neurogenesis takes place are described in the literature [12,27,28], the biomolecular changes accompanying this neuronal development are still unknown.

One previous study described biomolecular changes in the hippocampus in the early postnatal period and adulthood, which were monitored using FTIR spectroscopy [29]. They reported an accumulation of saturated and unsaturated lipids, as well as of compounds containing phosphate and carbonyl groups, during the juvenescence lifetime [30].

The aim of the present study was to use high-resolution SR-FTIR spectroscopy to investigate CA1, CA3 and granular zone (GZ) layers, and to show the difference from the SGZ where adult neurogenesis takes place. In addition, the goal was to uncover how the biomolecular composition of the SGZ in the adult hippocampus was investigated under different conditions of exposure to EE and to uncover the role of TnC in this process. Semiquantitative analysis revealed that, in the adult hippocampus, subtle changes in the distribution of main biomolecules and changes in their composition in hippocampal layers are present and that the SGZ is significantly different from CA3. Further investigation of the SGZ showed that exposure to EE leads to alterations in the composition of proteins and nucleic acids, with TNC in the extracellular matrix being needed for EE to exert its effects.

## 2. Materials and Methods

### 2.1. Animals

The first part of this study was performed on C57BL/6J mice, and the second part on constitutive tenascin-C-deficient (TnC KO) mice [31] and their wild-type littermates. Experiments were performed on two-month-old adult males. The number of experimental animals per group was 3–4 mice. The experimental procedures were in accordance with the NIH Guide for Care and Use of Laboratory Animals (1985) and the European Communities Council Directive (86/609/EEC). Experiments were approved by the local Ethics Committee. The authorization reference number is EK-BF-2020/02. All efforts were made to minimize animal suffering and to reduce the number of animals used.

### 2.2. Experimental Paradigm for Enriched Environment

In the current study, we used EE as an experimental paradigm to provide enhanced sensory, cognitive and motor stimuli. Males of TnC deficient mice and their wild-type littermates were housed in groups of eight animals per cage in larger cages (54 × 39 × 27 cm) containing various objects (boxes, ladders, balls, tunnels, tubes and running wheels) that differed in composition, shape, size, texture and brightness of color. The locations of the objects and the outlets for food and water were changed daily, and each week one object was replaced by another. This protocol was standardized within two cages used for these experiments. We started exposure to EE at the juvenile stage, around P21 [32] and housed the animals for 4 weeks with ad libitum access to food and water. For standard conditions (SC), four animals per cage were housed in conventional cages (32 × 20 × 13 cm) without objects but with ad libitum access to food and water at fixed positions. Mice were kept under a 12:12 h light/dark cycle.

### 2.3. Synchrotron-Based FTIR Spectroscopy and Imaging

#### 2.3.1. Tissue Preparation

Animals were sacrificed around two months of age. Three animals per group were used in this study. Animals were sacrificed by cervical dislocation and the brains were rapidly isolated and frozen at −80 °C. Coronal sections of the dorsal hippocampus were collected on a cryostat (CM1850, Leica Microsystems, Wetzlar, Germany) of 16 µm thickness and attached to 10 mm Ø × 0.5 mm CaF_2_ slides (Silson Ltd., Northampton, UK). The samples were freeze-dried for 24 h in the lyophilizer (TOPT-10C, Toption Instrument Co., Ltd., Hong Kong, China) and then slowly brought to room temperature and stored over silica gel before measurements. The tissues were imaged with a bright field microscope to investigate the tissue integrity and to determine the regions of interest.

#### 2.3.2. SR-FTIR Spectroscopy

Synchrotron-radiation light source was employed to collect individual spectra from different hippocampal layers. Samples were analyzed at the MIRAS BL01 beamline (ALBA Synchrotron, Barcelona, Spain) by using the 3000 Hyperion microscope coupled to a Vertex 70v spectrometer and liquid nitrogen-cooled mercury cadmium telluride (MCT) detector. IR spectra were collected in transmission mode using the 36x Schwarzschild objective/condenser and an aperture size of 12 × 12 μm. Approximately 40–50 spectra were collected from each region of interest in the 4000–800 cm^−1^ mid-infrared range at a spectral resolution of 4 cm^−1^ with 128 scans per spectrum. IR spectra were collected using Opus software (version 8.2, Bruker Company, Karlsruhe, Germany). OPUS software 7.5 was used for data acquisition and initial analysis. Quasar 9.1 (Orange-Spectroscopy, 0.8.0; Bioinformatics Laboratory, University of Ljubljana, Ljubljana, Slovenia) was used for subsequent analysis of spectra.

#### 2.3.3. Data Analysis

##### Correlation Analysis

Correlation analysis served as a key statistical approach to examine how distinct IR absorption bands relate to one another within the freeze-dried hippocampal tissue spectrum. Within the analyzed range of 3100–800 cm^−1^, we identified 15 distinct peaks/bands, each associated with a specific vibrational mode of a functional group. These selected peaks were then subjected to correlation analysis between experimental groups and the control group (SGZ), allowing us to discern patterns of peak clustering in relation to the control. By performing hierarchical cluster analysis (HCA) (see below) on Pearson’s correlation coefficients, we delineated main clusters that reflect the grouping tendencies of these functional group-related bands. We computed both Pearson’s correlation coefficients and their statistical significance using the rcorr (0.92) function from the Hmisc (5.1-3) package in R (4.4.0). The resulting correlation matrix was visualized through a correlogram generated by the corrplot function from the corrplot package, enabling a clearer interpretation of the clustering patterns and relationships among these IR spectral features [33].

##### Hierarchical Cluster Analysis

To uncover the intricate biomolecular relationships within distinct hippocampal layers or experimental groups, HCA was employed to compare the spectral profiles. Unlike in Correlation analysis (which compared pairwise only peak amplitudes), here, the spectra were compared for all four hippocampal layers and in a wider spectral range. In preparation for this similarity-based clustering, we applied a second derivative transformation and vector normalization to the raw spectra, following established procedures [34]. These preprocessing steps enhanced subtle differences and enabled a more reliable wavenumber-by-wavenumber comparison across all groups.

The entire spectral range was divided into three key intervals—lipid (3100–2800 cm^−1^), protein and ester (1800–1480 cm^−1^) and nucleic acid and carbohydrate (1270–800 cm^−1^)—to emphasize distinct biochemical signatures. To determine how clusters should form or split, Euclidean distances were calculated between the average spectra of these selected regions or experimental groups, yielding a matrix of pairwise differences. The resulting distance matrix was then visualized as a dendrogram, constructed using Ward’s linkage to reveal the clustering [35]. As clusters emerged along a heterogeneity scale, spectra with greater similarity converged into cohesive groups, thus highlighting underlying biochemical patterns among the hippocampal layers or experimental groups, respectively. Here and elsewhere in Data Analysis the Quasar 9.1 software was used.

##### Principal Component Analysis

Principal component analysis (PCA) was used as it provides dimensionality reduction for interpreting and visualizing the structure of high-dimensional data. Besides dimensionality reduction, PCA was also utilized for feature selection, in combination with other analysis techniques [35]. The term “PCA (factor) loadings” refers to the coefficients or weights associated with each original variable. They were useful in determining the relationship between the original variables (in this case, wavenumbers) and the new, reduced set of variables (the principal components—PCs). In this sense, large (either positive or negative) loadings indicate that a particular wavenumber is highly correlated with a principal component (in all experimental groups, as hypothesized). It also indicates that wavenumbers with comparable loadings contribute in an equivalent manner to the formation of the respective principal components. The sign and magnitude of the factor loadings may, therefore, provide information about the underlying structure and dynamics of the data (which could indicate that they are part of the same feature or process).

PCA was conducted on pre-processed spectral data encompassing key biomolecular domains: lipids (3100–2800 cm^−1^), protein and ester (1800–1480 cm^−1^) and nucleic acids and carbohydrate absorption (1270–800 cm^−1^). Before this analysis, raw spectra spanning 3100–800 cm^−1^ were refined using a second derivative transformation (Savitzky–Golay filter, 9-point smoothing, third-order polynomial) and vector normalization. By applying the second derivative, baseline contributions are effectively reduced, and broad, overlapping absorption bands become more distinctly resolved.

##### Linear Discriminant Analysis

Building upon the principal components obtained through PCA, a supervised classification approach—Linear discriminant analysis (LDA)—was then applied to enhance class separation and highlight subtle differences among the hippocampal layers or experimental groups, respectively [36,37]. By projecting the data in directions that maximize the separation between predefined classes, LDA addresses the limitations of PCA, which, as an unsupervised method, does not consider class differences [38]. The resulting PC-LDA score plots offer a clearer visual representation of how each layer’s or experimental group’s spectra diverge along the discriminant axes.

To further distinguish the factors underlying these separations, cluster vectors were derived from the PC-LDA loadings. These vectors detail the contribution of each PC to class differentiation, allowing us to pinpoint the specific spectral regions that are most influential in distinguishing the hippocampal layers or experimental groups, respectively [39].

##### Random Forest

In addition, we extend our supervised learning framework by incorporating a Random Forest (RF) classification approach to FTIR spectral data obtained from distinct brain regions and experimental conditions, aiming to refine our statistical modeling of class distinctions [40]. RF is an advanced ensemble machine learning method that constructs multiple decision trees during training and aggregates their outputs to produce a classification result. Beyond class prediction, it also assigns importance ranking to each feature—a particularly valuable attribute when dealing with FTIR data, where wavenumbers serve as features. Moreover, its resilience to overfitting and capacity to handle large feature sets make RF especially suitable for this type of spectral analysis.

Our RF models were trained using Quasar [40] on labeled spectral datasets, leveraging bootstrap aggregation (“bagging”) to enhance generalization and accuracy. By incorporating random subsets of both samples and features, the model reduces variance across its constituent trees and improves classification performance.

For the analysis, we employed second derivative spectra with vector normalization, focusing on three key spectral intervals: lipid (3100–2800 cm^−1^), protein and esters (1800–1480 cm^−1^) and nucleic acid and carbohydrate (1270–800 cm^−1^) regions. Within each region, RF identified the most influential wavenumbers—the specific spectral features that maximize the discriminative power between classes—thus providing insight into the biochemical differences underlying each classification.

##### Analysis of Integrated Band Area

For numerical comparisons of band area and peak positions of absorption bands associated with lipids, proteins and nucleic acids were analyzed. Raw spectra of different hippocampal layers were preprocessed by performing rubber band baseline correction and vector normalization. Afterward, functional groups were separately analyzed by calculating the band area based on assignments presented in Table 1.

### 2.4. Immunofluorescence

#### 2.4.1. Tissue Preparation and Immunostaining

Adult animals were anesthetized by intraperitoneal injection of ketamine (0.1 mL/100 g b.w.) and xylazine (0.01 mL/100 g b.w.). Transcardial perfusion was performed under anesthesia with PBS, followed by a 4% formaldehyde solution (Sigma-Aldrich) in PBS. Brains were postfixed in 4% formaldehyde solution for 48 h at 4 °C. Tissues were cryopreserved in 30% sucrose solution in 0.1 M phosphate buffer, pH 7.4 at 4 °C and frozen at −80 °C. Coronal sections of 30 µm thickness were cut with a cryostat (CM1850, Leica Microsystems, Wetzlar, Germany) and placed on slides (Superfrost Plus, Menzel Glaser, Braunschweig, Germany). After washing in PBS, the sections were incubated for 45 min at room temperature in the blocking solution containing 5% non-immune donkey serum and 0.2% Triton X-100 in PBS. Sections were then incubated with the primary rabbit anti-tenascin C antibody (TnC, 1:500, AbDSerotec, Duesseldorf, Germany) overnight at 4 °C. After washing in PBS, the sections were incubated with the secondary antibody donkey anti-rabbit AlexaFluor 488 (1:200, Invitrogen, Carlsbad, CA, USA). After washing in PBS at room temperature, slides were dried and covered with a coverslip mounting medium (Mowiol, Sigma Aldrich, St. Louis, Missouri, USA). For all immunohistochemical reactions, the sections from all experimental groups were processed in parallel. In addition, TO-PRO 3 (1:2000; Invitrogen, Thermo Fisher Scientific, Carlsbad, CA, USA, Cat. No. T3605) nuclear staining was used to identify the corresponding regions in all experimental groups.

#### 2.4.2. Image Acquisition

Two-dimensional images of immunostained sections were acquired with a confocal laser scanning microscope (Zeiss LSM 510; Oberkochen; Germany) where an Argon multiline laser (457, 478, 488 and 514 nm) was used for the excitation of Alexa Fluor 488 and a Helium-Neon laser (633 nm) for the excitation of TO-PRO3. Laser intensity, pinhole aperture, scanning speed, digital gain and offset were kept constant throughout the acquisition. After the acquisition, images were processed in ImageJ software (Version 1.54p).

### 2.5. Statistics

The significance of the differences among groups was calculated using the Kruskal–Wallis test with Dunn’s post hoc test in Graph Pad Prism 6 software (Northampton, MA, USA). Differences were considered significant with *p* < 0.05. To estimate the density of the TnC signal, we calculated the ratio of the mean TnC signal intensity and analyzed the area of the SGZ. We used the parametric Student’s *t*-test (with Welch’s correction) for statistical analysis with Graph Pad Prism 6 software (Northampton, MA, USA). Power analysis was performed on the data regarding TnC expression to validate the number of sections per slice sufficient for signal quantification.

## 3. Results

### 3.1. Correlation Analysis of FTIR Spectral Data Between Hippocampal Layers

**Figure 1:**
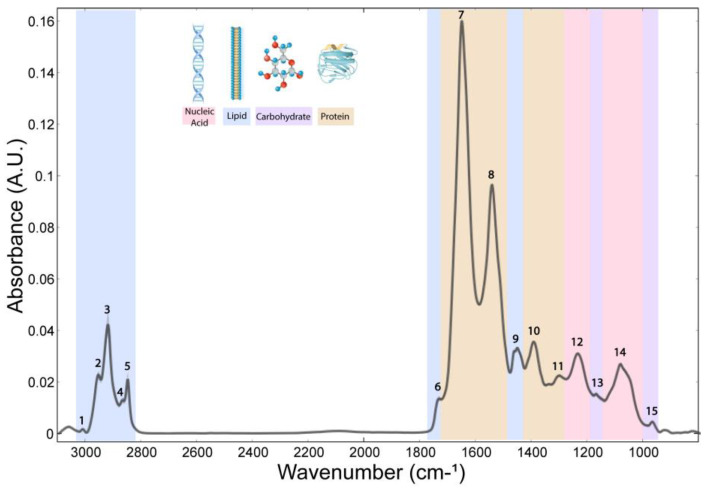
Average FTIR spectra acquired in hippocampal layers of freeze-dried tissue. The numbers indicate peaks assigned to a specific chemical bond (related to Table 1) and classified into main biomolecular compounds—lipids, proteins and ester, nucleic acids and carbohydrates indicated by band colors.

**Figure 2:**
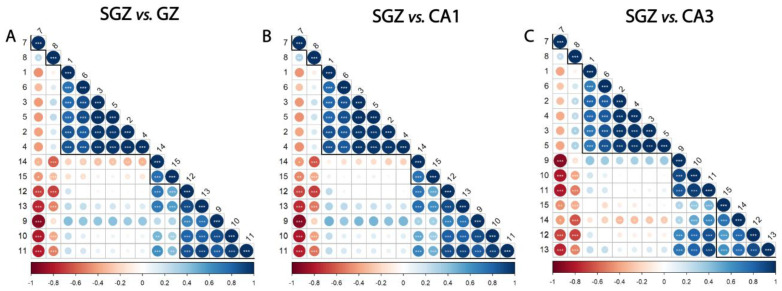
Subgranular zone (SGZ) and CA3 layers show distinctive clustering patterns. Correlation analysis of (**A**) SGZ vs. granular zone (GZ), (**B**) SGZ vs. CA1, (**C**) SGZ vs. CA3. Peaks are grouped and ordered by hierarchical clustering with Ward’s linkage. Numbers on the axes denote the peaks assigned to biomolecules, using the same annotation as in Table 1. The degree of pairwise correlation, as indicated by Pearson’s correlation coefficient, is represented through a combination of a color gradient and varying circle sizes, with the colors reflecting the nature of the correlation (positive or negative) and the circle size reflecting the value of the coefficient. The significance of the correlation is indicated by the label * inside the circle (* *p* ≤ 0.05, ** *p* ≤ 0.01, *** *p* ≤ 0.001). Bold lines indicate clusters.

The whole spectrum (3100–800 cm^−1^) of the freeze-dried hippocampus tissue was characterized by 15 bands (Figure 1), which were assigned to specific vibrational modes of functional groups (Table 1). The SGZ, GZ, CA1 and CA3 layers contained all 15 peaks, so the variation patterns at these specific bands within the spectra were analyzed. Peak correlation analysis was performed between pairs of data, always using the SGZ as a control group (Figure 2). Consequently, peaks were grouped into clusters based on Pearson’s correlation coefficient. The SGZ vs. GZ and SGZ vs. CA1 comparison showed the same pattern of clustering, while the SGZ vs. CA3 comparison pointed to a different clustering of peaks originating from the nucleic acid absorption region of the spectra. Namely, five main clusters were found with significant Pearson’s correlation coefficients greater than 0.58 for all band pairs (Figure 2). Clusters 1 and 2 (counting from the top of the matrix) were comprised of single peaks 7 and 8, corresponding to Amide I and Amide II bands, respectively. The third cluster consisted of peaks 1 to 6, all assigned to lipids. The main difference in the correlation plots was observed in the clustering of peaks 12 and 13, primarily related to the nucleic acid and carbohydrate content. More specifically, in the case of the SGZ vs. GZ and SGZ vs. CA1 comparisons, peaks 12 and 13 were clustered together, with peaks 9, 10 and 11 assigned to proteins. However, in the SGZ vs. CA3 comparison, peaks 12 and 13 were clustered together with peaks 14 and 15 that originate from vibrations from nucleic acid bonds, which indicates that nucleic acids contribute to a greater extent.

The correlation analysis showed that the peaks originating from proteins and lipids always formed the same clusters, while the differences between the spectra were in the fingerprint range (1400–800 cm^−1^) and related to the peaks associated with nucleic acids. Comparison of the SGZ with GZ and CA1 resulted in the same clustering of peaks, indicating the similarities of the spectra. The specificities in the CA3 spectrum led to different clustering in the SGZ vs. CA3 comparison in the nucleic acid region, pointing out that the CA3 is the most different layer from the SGZ. 

### 3.2. Similarity of Hippocampal Layers

**Figure 3:**
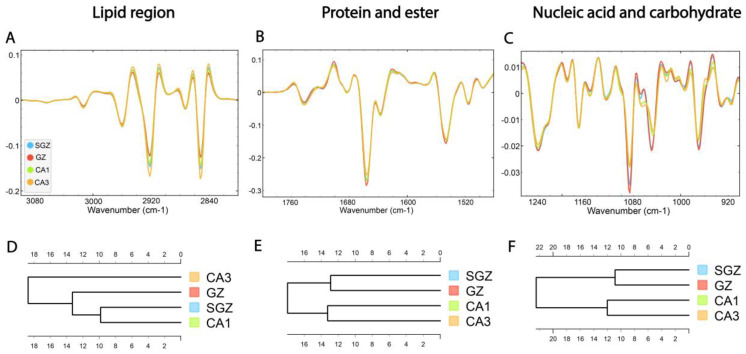
The average FTIR spectra of freeze-dried hippocampus tissue in the SGZ, GZ, CA1 and CA3 layers. (**A**) Lipid spectral region 3100–2800 cm^−1^, (**B**) protein and ester 1800–1480 cm^−1^ and (**C**) nucleic acid and carbohydrate 1270–800 cm^−1^. Hierarchical clustering of SGZ, GZ, CA1 and CA3 layers using Euclidian’s distance based on their average spectra in the lipid (**D**), protein (**E**) and nucleic acid (**F**) regions.

Following correlation analysis, HCA was conducted on the processed spectra. The dendrograms were constructed based on average spectra of selected spectral regions—lipid (3100–2800 cm^−1^), protein and ester (1800–1480 cm^−1^) and nucleic acid and carbohydrate (1270–800 cm^−1^)—originating from different hippocampal layers (Figure 3A–C). The dendrogram based on spectra acquired in the lipid region revealed that the SGZ has the highest degree of similarity with CA1 (Figure 3D). In the protein and nucleic acids region, the same clustering pattern was observed. The SGZ and GZ layers were grouped together into the first main cluster, and CA1 and CA3 formed the second one (Figure 3E,F).

In summary, depending on the biomolecular spectral origin, hierarchical clustering analysis revealed that the SGZ closely resembled the GZ in protein and nucleic acid composition and CA1 in lipid composition. On the other hand, CA3 consistently did not form a cluster with the SGZ.

### 3.3. Biomolecular Characterization of Hippocampal Layers

Further analysis focused on examining the differences between the layers. It is important to highlight that the spectra of the SGZ, GZ, CA1 and CA3 show high qualitative similarity in terms of the presence of specific bands, so the variability of the data originated from the differences detected in the position and intensity of specific bands.

PCA analysis was supplemented with LDA in order to maximize the differences between predefined classes, thereby improving the visual separation between the groups [37]. Also, RF classification was used to understand which bands are the most important for classification.

**Figure 4:**
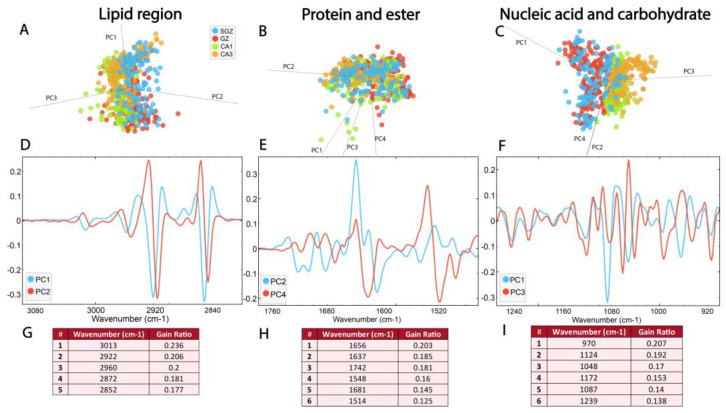
The SGZ, GZ, CA1 and CA3 layers differ in biomolecular composition, most prominently in the nucleic acid region. The PCA-LDA score plots with PCs show the distribution of spectral data of different hippocampal regions in the (**A**) lipid spectral region 3100–2800 cm^−1^, (**B**) protein and ester region 1800–1480 cm^−1^ and (**C**) nucleic acid and carbohydrates region 1270–800 cm^−1^. The loading plots (**D**–**F**) show the contribution of individual absorbance to the PCAs of the selected PCs. The RF classification (**G**–**I**) shows the ranking of the peaks that contribute the most to the data classification.

The PCA-LDA score plots showed that the first three principal components, which accounted for 98% of the variance, contributed to the separation of the data within the lipid region (3100–2800 cm^−1^) (Figure 4A, Supplementary Figure S1A). The loadings plot showed a contribution of ν_s_ and ν_as_CH_2_ bands located on PC1 and PC2 (Figure 4A), as well as CH_3_ and C=C bands. It also pointed out the minima at ∼2922 cm^−1^, ∼2852 cm^−1^ and ∼2956 cm^−1^ for PC1 (and PC2 as well) and a maximum at ∼2872 cm^−1^ (Figure 4D, Supplementary Table S1). In agreement with these results, RF classification confirmed that bands originating from saturated lipids were the most important features for the origin of the biomolecular differences in hippocampal structural formations. Specifically, CH_2_ bands received the highest feature importance ranking in the RF classification, followed by CH_3_ and C=C bending bands. (Figure 4G).

Within the protein and ester region, bands originating from carbonyl groups, as well as Amide I and II, were detected. Figure 4B illustrates the distribution of the data in this region (1800–1480 cm^−1^), as described by the first four principal components, which account for 97% of the data variance. The average spectra of the second derivatives and the score plots exhibit prominent overlap (Figure 3 and Figure 4B, Supplementary Figure S1B). However, the loadings plots revealed differences between four hippocampal layers in the secondary protein structure, identifying peak maxima for PC2 at ~1656 cm^−1^, ~1637 cm^−1^, ~1514 cm^−1^ and ~1681 cm^−1^, and for PC4 at ~1742 cm^−1^ and ~1548 cm^−1^ (Figure 4E, Supplementary Table S1). Furthermore, RF classification ranked peaks at ~1656 cm^−1^ and ~1637 cm^−1^ from the Amide I band, corresponding to α-helix and β-sheet, respectively, as the most important features explaining the differences among the layers (Figure 4H).

The most prominent differences between the four hippocampal layers were detected in the nucleic acid region (1270–800 cm^−1^), where proteins and carbohydrates also contribute to absorption. The PCA-LDA score plots demonstrated the separation of the layers based on four PCs, which account for 97% of the data variance (Figure 4C, Supplementary Figure S1C). Analysis of individual absorbance contributions to PCA showed relevant vibrational bands at ~1086 cm^−1^, ~1046 cm^−1^, ~970 cm^−1^, ~1172 cm^−1^ and ~1243, cm^−1^, observed as minima on PC1 (Figure 4F, Supplementary Table S1). Furthermore, RF rankings indicated that peaks at ~1086 cm^−1^ and ~1046 cm^−1^ from ν_s_ phosphate band—corresponding to nucleic acid and carbohydrate—and ~970 cm^−1^—corresponding to nucleic acids—have the largest contribution to classification (Figure 4I).

In essence, the main peaks ranked by RF classification correspond to the most significant peaks observed in the PCA loadings plot (Figure 4G–I). As our results demonstrate, multivariate analysis (Figure 3 and Figure 4) successfully separated the SGZ, GZ, CA1 and CA3 hippocampal layers based on their biomolecular fingerprints and confirmed the feasibility of detecting subtle differences in the hippocampal layers using SR-FTIR.

### 3.4. Integrated Band Area Analysis of the Hippocampal Biomolecular Content

When significant spectral differences are present, the integrated band area method is commonly used for analysis, enabling straightforward interpretation. We thus examined integrated band areas to assess relative changes in biomolecule content within hippocampal layers. This analysis aimed to compare our findings with those of Chwiej and colleagues in order to validate SR-FTIR spectroscopy for identifying hippocampal layers [29].

We analyzed the integrated band area of the absorption bands in the 2800–3100 cm^−1^ range to detect changes in lipid content. The C=C band (~3014 cm^−1^) provides information about the unsaturated lipid content. Lipid peroxidation primarily takes place at the double bond sites of polyunsaturated fatty acids, causing a decrease in the amount of unsaturated lipids. As seen from Table 2A, there are significant differences in the unsaturated lipid content in different layers of the hippocampus. Nevertheless, the SGZ and CA1 layers show no significant difference in C=C band content, which is in accordance with the HCA results that showed the greatest similarity between these two layers in the lipid region (3100–2800 cm^−1^). The same trend was observed in the case of the carbonyl ester (~1742 cm^−1^) (Table 2B), which referred to the content of carbonyl groups in triglycerides or cholesterol esters in this group.

Nucleic acids mainly absorb in the spectral area between 1270 and 800 cm^−1^, with a contribution from phospholipids, with the changes in the shape, position or intensity of associated bands providing information about the structural integrity of nucleic acids. This study reports minor changes in the band area of ν_s_ and ν_as_ PO_2_^−^, C-O deoxyribose bends and significant changes in the position of ν_s_ PO_2_^−^ band (Table 2C–F).

### 3.5. Tenascin C in the Adult SGZ and the Effect of Enriched Environment

**Figure 5:**
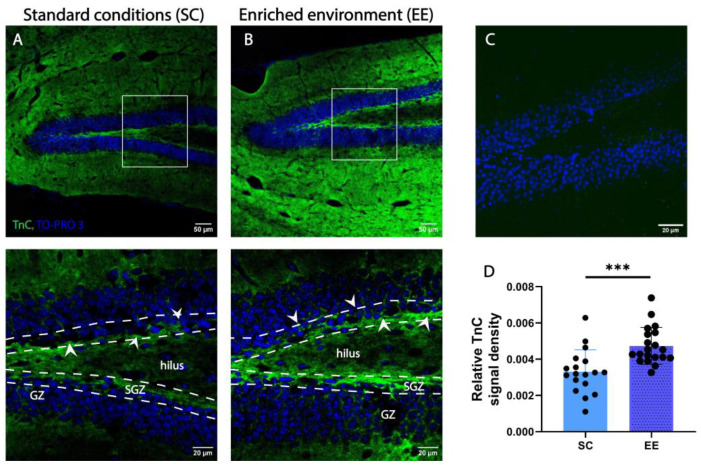
An enriched environment (EE) increases the expression of TnC in the SGZ of adult wild-type mice. (**A**,**B**) Representative confocal images of immunofluorescence staining for extracellular matrix glycoprotein TnC (green) and TO-PRO3 as nuclear staining (blue) in the dentate gyrus (DG) of wild-type mice housed in standard conditions (SCs) ((**A**) white rectangle in the top panel indicates the area presented in the bottom panel) and EE ((**B**) top panel indicates the area presented in the bottom panel). Interrupted white lines delineate the separation of SGZ from the hilus and GZ layers. Note the localization of TnC around the SGZ indicated by white arrows. Scale bars 50 µm (left) and 20 µm (right). (**C**) Immunostaining for TnC in TnC-/- mice. (**D**) Quantification of the pixel density for TnC per section (n = 3–4 mice/group; each data point represents one microscopy section per slice). Statistical difference was determined by Student’s *t*-test. Bars represent normalized mean ± SD. *** *p* ≤ 0.01, (*p* = 0.0004). GZ—granular zone.

TnC expression has been reported to decrease with age and to be restricted to areas of functional plasticity and active neurogenesis [42]. To localize the expression of TnC in the DG of adult mice, we used immunostaining for TnC and TO-PRO 3 as counterstaining. Figure 5 shows an immunofluorescence image depicting the DG structure with successive layers GZ, SGZ and hilus (note interrupted white lines in Figure 5A,B bottom). We found that TnC expression in the DG hilus is localized as a dense formation along the GZ but also around the granule cells in the first few cell layers of the GZ (arrows in Figure 5). TnC immunostaining was undetectable in adult TnC KO mice (Figure 5C), confirming the specificity of the antibody.

To address the question of whether EE affects the expression of TnC in the DG of adult mice, we compared samples of wild-type mice housed in two different conditions—EE and SC. After comparing the immunostained slides from EE and SC, we found that EE had a positive effect on the expression of TnC in the SGZ of adult wild-type mice by increasing the density of the TnC signal (0.0047 ± 0.0012 and 0.0033 ± 0.0012, respectively; Figure 5D).

### 3.6. Comparison of the Biomolecular Composition of the SGZ in Different Experimental Conditions and the Role of TnC

**Figure 6:**
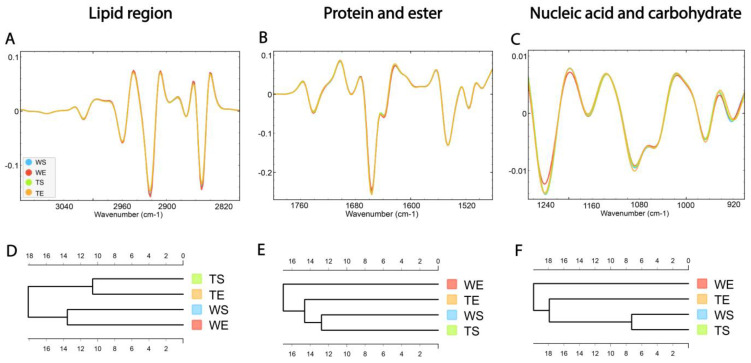
The average FTIR spectra of freeze-dried hippocampus tissue of the SGZ in (**A**) lipid spectral region 3100–2800 cm^−1^, (**B**) protein and ester 1800–1480 cm^−1^ and (**C**) nucleic acid and carbohydrate 1270–800 cm^−1^. Hierarchical clustering of different experimental groups using Euclidian distance based on their average spectra are presented in the lipid (**D**), protein and ester (**E**) and nucleic acid and carbohydrate (**F**) regions. Wild-type mice housed in standard conditions—WS, wild-type mice housed in an enriched environment—WE, TnC-deficient mice housed in standard conditions—TS, TnC-deficient mice housed in an enriched environment—TE.

HCA was conducted on the processed spectra and the dendrograms were constructed based on averaged spectra of selected spectral regions—lipid (3100–2800 cm^−1^), protein and ester (1800–1480 cm^−1^) and nucleic acid and carbohydrate (1270–800 cm^−1^) originating from different experimental groups—for wild-type mice housed in standard conditions (WS), wild type mice housed in an enriched environment (WE), TnC-deficient mice housed in standard conditions (TS) and TnC-deficient mice housed in an enriched environment (TE) (Figure 6A–C).

The dendrogram based on spectra acquired in the lipid region revealed that the WS and WE groups formed one cluster, while TS and TE formed the other one (Figure 6D). The effect of the genotype was apparent regardless of the effect of the environment. In the protein and ester (1800–1480 cm^−1^) and nucleic acid and carbohydrate regions (1270–900 cm^−1^), the WE group formed a separate cluster, indicating the influence of EE on the composition of these two groups of molecules. Also, the TE group was clustered closer to the group of WS and TS than to WE (Figure 6E,F).

### 3.7. Biomolecular Profile of the SGZ Depends on the Environmental Conditions and Expression of TnC

**Figure 7:**
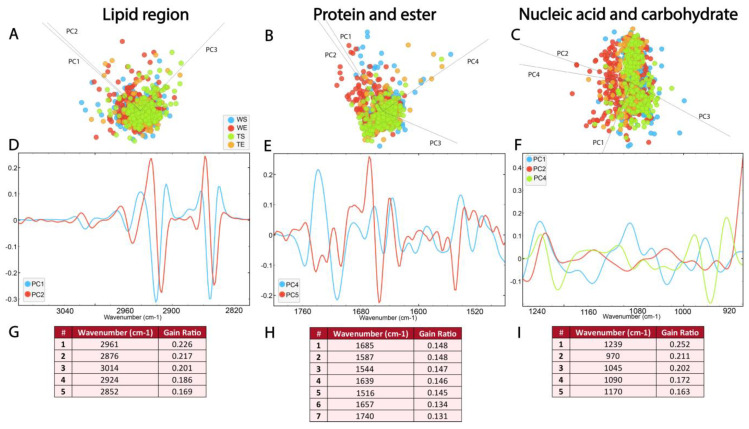
The absence of TnC leads to subtle changes in the FTIR spectra of the adult SGZ. PCA-LDA score plots with PCs (describing ~99% of variance) showing the distribution of spectral data of different experimental groups (see Figure 6) in the (**A**) lipid 3100–2800 cm^−1^, (**B**) protein and ester 1800–1480 cm^−1^ and (**C**) nucleic acids and carbohydrate regions 1270–900 cm^−1^. Loading plots (**D**–**F**) show the contribution of individual absorbance to the PCAs of the selected PCs. RF classification (**G**–**I**) shows the ranking of the peaks that contribute the most to the data classification.

Figure 7 represents the biomolecular FTIR spectra of the SGZ in different experimental groups. As previously mentioned, in the case of FTIR characterization of hippocampal layers, the spectra acquired in the SGZ of different experimental groups exhibited similar FTIR signatures, with all previously detected peaks present. Therefore, the analyzed differences involved subtle variations in band intensities, which were examined and distinguished using multivariate analysis methods, specifically, PCA-LDA and RF classification.

PCA-LDA score plots illustrate the distribution of the data within the lipid region (3100–2800 cm^−1^) across different experimental groups, with three PCs accounting for 96% of the variance (Figure 7A, Supplementary Figure S1D). The loadings plot showed a contribution of ν_s_ and ν_as_CH_2_ bands in PC1 and PC2 (Figure 7A), as well as CH_3_ and C=C bands. The loadings plot pointed to minima at ∼2922 cm^−1^, ∼2852 cm^−1^, ∼2956 cm^−1^ and ∼2872 cm^−1^ for PC1 and PC2 (Figure 7D, Supplementary Table S2). The RF classification showed that the ν_as_ CH_3_ and C=C absorbance bands had the highest rank, followed by the ν_s_ and ν_as_ CH_2_ bands (Figure 7G). The differences between experimental groups in the lipid region, which were recorded as minima in the loadings plot and as peaks with the highest ranks in RF classification, can, therefore, be attributed to the ν_as_ CH_3_ and C=C bands.

The spectral range between 1800 and 1480 cm^−1^ was analyzed to detect changes in ester and protein content. As illustrated in Figure 7B, the average second derivative spectra in the protein and ester regions exhibit prominent overlap (Figure 7B, Supplementary Figure S1E). The PCA-LDA score plots illustrate the distribution of the data, with four PCs accounting for 96% of the variance. The loadings plot showed the differences in the secondary protein structure, and the loading plots pointed to maximums in PC4 at ~1742 cm^−1^ and ~1637 cm^−1^, and minimums at ~1681 cm^−1^, ~1656 cm^−1^, ~1548 cm^−1^ and ~1516 cm^−1^ (Figure 7E, Supplementary Table S2). Interestingly, peaks at ~1685 cm^−1^, ~1639 cm^−1^ and 1516 cm^−1^, corresponding to β-turn, β-sheet and tyrosine amino acid residues, were ranked as the most significant for the RF classification (Figure 7H). In addition to the presence of bands originating from the α-helix, the bands originating from the β-sheet stood out as the most important features for the differences between the experimental groups.

In the PCA-LDA analysis, the distribution of the data in the nucleic acid region (1270–900 cm^−1^) is represented with four PCs accounting for 95% of the variance (Figure 7C, Supplementary Figure S1F). Analysis of the contribution of individual absorbances to the PCA in the nucleic acid spectral region (1270–900 cm^−1^) revealed prominent differences in vibrational bands at ~1239, cm^−1^ ~1090 cm^−1^, ~1045 cm^−1^ and ~970 cm^−1^, which were observed as maxima on PC1 (Figure 7E, Supplementary Table S2). RF rankings indicated that peaks at ~1239 cm^−1^, ~1045 cm^−1^ and ~970 cm^−1^, all corresponding to nucleic acid, make the largest contribution to classification (Figure 7I). This indicates the significance of the composition and organization of the nucleic acids for the generation of differences between experimental groups.

## 4. Discussion

The present study succeeded in using SR-FTIR spectroscopy as a novel approach to detect differences in biomolecular composition between hippocampal layers that are present during development and persist, to a reduced, subtle extent, in adulthood. The differences detected in the adult hippocampus between the SGZ, GZ, CA1 and CA3 layers corroborated the subtle differences described in [29]. Moreover, using the high-resolution SR-FTIR spectroscopy enabled the characterization of the SGZ layer, despite its subtle, thin cellular structure between the hilus and granule cells.

This study is in line with previous FTIR spectroscopy studies that successfully characterized the hippocampus after traumatic brain injury [10], systematic inflammation [43], or in the case of underlying pathology [9,41], where drastic changes were easily detectable with FTIR spectroscopy. Also, the technique was used to study physiological conditions, such as aging and development. It was shown that the changes in the biomolecular composition of the hippocampal layers are significant during development, while they decrease during aging, as do the differences between the layers [29]. A time point at around two months of age was labeled as critical when the initial rapid changes slowed down, leading to a more stable biochemical environment. This stage is marked by the stabilization of protein and lipid levels and by a decrease in the difference among regions, indicating that the key phases of postnatal brain development have been successfully completed. In other words, this is a time point when adult neurogenesis exhibits a drastic decrease in rate in rodents [30].

Further investigation of the hippocampus of two-month-old mice showed similarity among the layers regarding the presence of all peaks, which can be attributed to the common building molecules of the pyramidal and granular layers. The differences were subtle but detectable and can be related to different metabolic and physiological processes taking place in these layers. Namely, CA3 was the most distinctive, and it was never clustered together with the SGZ (Figure 2, Table 2, Figure 3). The biggest differences were in the nucleic acid region (1270–800 cm^−1^), with the most prominent contributions coming from the ν_as_ PO_2_^−^ and C-O deoxyribose bands, which were assigned to phosphodiester and deoxyribose stretching in the DNA, pointing out differences in DNA organization and structure in different hippocampal layers (Dučić et al., 2023 [31]). The correlation analysis showed that there is a different clustering of peaks between SGZ and CA3 only in this spectral region (Figure 2; peaks 12 and 13). The above results are of particular biological significance since they demonstrate the dominant role of the SGZ in adult neurogenesis, while the observed changes in DNA/RNA structuring may indicate epigenetic biomolecular modifications.

Based on PCA-LDA, the layers SGZ and GZ and CA1 and CA3 were grouped together (Figure 3E, Figure 4F and Figure S1). The results revealed a similar content of nucleic acids in the SGZ and GZ, as well as in CA1 and CA3 (Table 2D), which may be related to the density of cells in these layers. Besides the apparent difference in the type of neurons present in these layers, adult neurogenesis and accompanying processes are exclusively related to the SGZ and GZ layers. All pyramidal neurons are generated during development, while granular neurons are continuously generated in the SGZ during adulthood. Due to the intense changes during development, the differences between these layers in biomolecular composition are more pronounced compared to the adult period, when a certain biomolecular balance is established. It was observed that, during development, there is an accumulation of biomolecular components in most cell layers of the hippocampus, except in the granular layer in the case of phosphate compounds [29]. This supports the hypothesis that the biomolecular changes in the whole hippocampus are closely related to the rate of neurogenesis that changes with age.

Differences in the composition of lipids were mostly connected to the ν_s_ CH_2_ and ν_as_ CH_2_ bands assigned to saturated lipids (Figure 4G). The content of C=C and carbonyl bonds present in unsaturated lipids, which are considered parameters that indicate the oxidative state of the cell, were highest in CA3 (Table 2A,B). CA3 pyramidal cells are relatively resistant to ischemic damage and cell death compared to those of CA1 [44] due to the fact that CA1 pyramidal cells are more susceptible to ischemic damage because of their high energy dependence. Therefore, the content of double bonds in CA3 can be related to balanced energy consumption and the generation of free radicals that cause oxidative stress. Additionally, the content of double bonds in the SGZ was increased compared to in the GZ (Table 2A,B). Other studies reported higher levels of oxidative stress parameters in the SGZ than in the GZ, indicating localized hypoxia in the SGZ [27]. They connected metabolic conditions in the SGZ with a critical role of this structure in the early and activity-independent survival of adult hippocampal newborn cells.

The changes in Amide I indicate subtle differences between hippocampal layers in terms of protein secondary structure and organization (Figure 4B). However, in cases of injury or pathology, such as neurodegeneration, a large decrease in total lipid and total protein content was documented [10,45]. The present study was performed under physiological conditions, so subtle differences were expected and confirmed.

In order to check the biomolecular composition of the SGZ under altered conditions, we followed, using SR-FTIR spectroscopy, the effects of EE with or in the absence of TnC. Because the SGZ contains cells at different developmental stages, it has a heterogeneous cell composition [46]. The use of SR-FTIR spectroscopy involves collecting information about the biochemical composition of bulk tissue and providing general information about the biomolecules present. Exposure to EE induces apparent metabolic changes at the cellular level, as revealed by various omics techniques that offer high chemical resolution and selective detection of specific compounds [28,47]. Exposure to EE leads to the altered expression of many different groups of genes, especially those related to synaptic plasticity [48]. Even though, as previously emphasized, the use of SR-FTIR spectroscopy involves collecting information from the bulk tissue and providing general information, the differences present here could also be revealed with the HCA approach, revealing the changes induced in the above experimental conditions.

As for TnC, its localization and expression in the SGZ and its increase after exposure to EE were revealed. Expression of TnC in the hippocampus was already described under conditions of long-term potentiation [49]. That study reported the expression of TnC in the strata oriens and radiatum of the CA1, stratum oriens of the CA3 and within a narrow area at the inner surface of the granule cell layer in the dentate gyrus. Also, a more recent study investigated a single-cell transcriptome of NSCs from the dentate gyrus and showed that the TnC gene is among the SGZ-enriched expression patterns [50]. The present study confirms the accumulation of TnC between the hilus and granular cell layer and, in addition, the presence of TnC, particularly within the SGZ (Figure 5). The close association of TnC and the cells in the SGZ suggests that this regulatory protein contributes to NSC maintenance in the local microenvironment. We also showed that exposure to EE leads to increased expression of TnC in the SGZ. A positive effect of EE on TnC expression was previously reported in the deep cerebellar nuclei, a brain region that, in addition to the hippocampus, takes part in neuronal plasticity [51]. The SR-FTIR spectroscopy data revealed that the effects of EE on protein and nucleic acid content in the SGZ could be correlated with the congruent increased expression of TnC. The present study, however, reports no effect of TnC itself on the content of proteins and nucleic acids in the SGZ. Nevertheless, under the influence of EE, in which the expression of TnC is increased, an effect is observed. Considering the way in which the WE group differs from the other experimental groups, as revealed by HCA, one can discuss the effect of EE on the composition of nucleic acids. The largest differences in the nucleic acid region, as revealed by SR-FTIR spectroscopy, were related to ν_as_ PO_2_^−^ and ν_s_ PO_2_^−^ (Figure 7I), suggesting differences in DNA expression and probably epigenetic modification [34]. This finding is congruent with the positive influence of EE on the survival of new cells in neurogenesis [23]. Since a minimal duration of EE exposure of three weeks is necessary to induce neurogenesis [52], we took a similar approach in this study to be able to detect underlying biomolecular changes. Still, the redistribution of genetic material and cellular reorganization during cell division in the tissue can lead to further subtle and transient spectral changes.

In general, differences in the protein region are observed in the secondary structures of proteins. Peaks related to both α-helix and β-sheets were located on the same PC (Figure 7E). However, a more detailed analysis showed a significant contribution of the bands associated specifically with the β-sheets. This finding is particularly intriguing, given that the β-sheets are typically associated with protein aggregation in neurodegeneration. This assertion can be further supported by the presence of a tyrosine band, which is also associated with oxidative stress [53]. Interesting findings have already been published with FTIR spectroscopy, such as the dominance of α-helix bands in the spectra of human mesenchymal stem cells during cell differentiation [6]. Still, results from this study could not be related to specific cell processes due to the complexity of the tissue samples investigated.

The above findings are of particular clinical relevance in hippocampal pathology. For instance, the hippocampus is a crucial region in bipolar disorder, and hippocampal neurogenesis has been proposed as a target of early interventions in individuals at high risk for bipolar disorder, such as the offspring of patients. Indeed, consistent findings from multiple fMRI studies have demonstrated disruptions in hippocampal brain networks not only in bipolar disorder patients but also in the offspring of patients [54].

In addition, differences in lipid structures also contribute to the distinguished profile of the SGZ, while the ν_as_ CH_3_ and C=C bands present the most important of the observed changes (Figure 7I). Also, HCA showed that the two genotypes (wild type and TnC KO) were similar regardless of the environmental influence (EE or SC). The transition between immature and mature neurons is accompanied by changes in their lipid metabolism, increasing de novo lipogenesis and decreasing β-oxidation [55]. Therefore, the potential role of TnC can be related to the regulation of this transitional process. As previously reported, TnC interacts with lipid rafts of astrocytes and neurons of both neonatal and adult mice, enabling signal transduction through membrane-associated proteins [56]. A later study described a functional consequence of binding TnC to lipid rafts in oligodendrocytes in rat cell cultures, defining the signaling pathway involved in the differentiation of oligodendrocyte precursors [57]. 

## 5. Conclusions

Based on previous research, FTIR spectroscopy combined with multivariate analysis of spectral data provides a preliminary understanding of the screening of metabolic changes in proteins, lipids and carbohydrates. In addition, the comprehensive approach of this method is useful before employing other more targeted methods.

This study showed that there are discrete differences between the layers in the adult hippocampus that represent residual changes largely expressed during development. These differences are mainly present in the nucleic acid region. The rate of changes in the hippocampal layers follows the rate of neurogenesis, with these effects being enhanced under the influence of EE. It was shown that the above processes were accompanied by corresponding changes in the protein and nucleic acid composition, which were dependent on the presence of TnC. The absence of TnC resulted in changes related to β-sheets and DNA bonds. These alterations point towards a rearrangement in the protein secondary structure and metabolism of the nucleic acid compounds.

However, additional studies are needed to better understand the molecular responses in the SGZ in the absence of TnC. Immunohistochemical analysis of specific cell types that participate in adult neurogenesis could address the limitations of the current study and better characterize the specific role of TnC. Also, FTIR spectroscopy shows potential as a complementary method to conventional methods, such as FACS, when combined with other complementary approaches, such as proteomics and lipidomics, and it should provide a deeper understanding of the biochemical changes that accompany cell development in the SGZ.

Finally, future research should be directed to further understand the clinical relevance of the applied method and the observed molecular markers for a better understanding of the role of hippocampal neurogenesis in the pathophysiology and therapy of diseases such as Alzheimer’s, Parkinson’s or Huntington’s.

## Figures and Tables

**Table 1:** Assignment of spectral bands to molecular vibrations of functional groups and biochemical compounds, based on similar biological systems described in the literature. The wavenumbers are assigned to main biomolecular compounds—lipids, proteins and ester, nucleic acids and carbohydrates indicated by table background colors (related to band colors in Figure 1). Note for table ν—bond stretch; s—symmetric vibration; as—asymmetric vibration; δ—bending vibration. Color code as in Figure 1.

Band	Wavenumber (cm^−1^)	Vibrational Modes and Functional Groups	Main Biochemical Compounds	Other Biochemical Compounds	References
1	3012	νC=C	Unsaturated fatty acids	Aromatics	[30,41]
2	2957	ν_as_CH_3_	Saturated lipids	Proteins, carbohydrates, nucleic acids	[11,41]
3	2924	ν_as_CH_2_	Saturated lipids	Proteins, carbohydrates, nucleic acids	[11,41]
4	2872	ν_s_CH_3_	Saturated lipids		[41]
5	2852	ν_s_CH_2_	Saturated lipids		[9,41]
6	1739	νC=O	Triglycerides, cholesterol esters	Lipids, phospholipids	[9,30]
7	1657	Amide I	Proteins	Unsaturated fatty acids	[30,41]
8	1544	Amide II	Proteins	Aromatics	[41]
9	1468	δCH_2_	Lipids	Proteins	[6]
10	1393	n_s_COO^−^	Amino acids and fatty acids	Other carboxylates	[41]
11	1304	Amide III	Proteins		[41]
12	1241	ν_as_PO^2−^	Nucleic acids	Phospholipids	[30]
13	1171	ν_as_CO-O-C	Carbohydrates	Proteins	[6]
14	1086	ν_s_PO^2−^	Nucleic acids	Phospholipids	[30]
15	967	C-O deoxyribose, C-C DNA	Carbohydrates	Nucleic acid	[6]

**Table 2:** Integrated band area median values of spectra of SGZ, GZ, CA1 and CA3 layers at specific wavelength ranges related to the bond vibrations in specific molecules. Kruskal–Wallis, (* *p* ≤ 0.05, ** *p* ≤ 0.01, *** *p* ≤ 0.001, ns—nonsignificant). Also, see Figure 1. ν—bond stretch; s—symmetric vibration; as—asymmetric vibration.

**(A)**				
**C=C**				
	**Mean rank difference**	**Signifficance**	***p*** **-value**	**Z score**
**GZ vs. SGZ**	−65.4	**	0.006	3.31
**GZ vs. CA1**	−93.7	***	<0.001	5.12
**GZ vs. CA3**	−154	***	<0.001	8.02
**SGZ vs. CA1**	−28.3	ns	0.961	1.4
**SGZ vs. CA3**	−88.9	***	<0.001	4.23
**CA1 vs. CA3**	−60.6	*	0.012	3.08
**(B)**				
**C=O**				
	**Mean rank difference**	**Signifficance**	***p*** **-value**	**Z score**
**GZ vs. SGZ**	−53	*	0.044	2.68
**GZ vs. CA1**	−90.8	***	<0.001	4.96
**GZ vs. CA3**	−162	***	<0.001	8.44
**SGZ vs. CA1**	−37.8	ns	0.365	1.87
**SGZ vs. CA3**	−109	***	<0.001	5.2
**CA1 vs. CA3**	−71.6	**	0.002	3.64
**(C)**				
**ν** **_as _** **PO_2_^−^**				
	**Mean rank difference**	**Signifficance**	***p*** **-value**	**Z score**
**GZ vs. SGZ**	−45.1	ns	0.134	2.29
**GZ vs. CA1**	−27.3	ns	0.816	1.49
**GZ vs. CA3**	−70.6	**	0.001	3.67
**SGZ vs. CA1**	17.9	ns	>0.999	0.886
**SGZ vs. CA3**	−25.5	ns	>0.999	1.21
**CA1 vs. CA3**	−43.4	ns	0.164	2.21
**(D)**				
**C-O deoxyribose**				
	**Mean rank difference**	**Signifficance**	***p*** **-value**	**Z score**
**GZ vs. SGZ**	−6.7	ns	>0.999	0.339
**GZ vs. CA1**	163	***	<0.001	8.94
**GZ vs. CA3**	123	***	<0.001	6.37
**SGZ vs. CA1**	170	***	<0.001	8.44
**SGZ vs. CA3**	129	***	<0.001	6.15
**CA1 vs. CA3**	−40.8	ns	0.227	2.08
**(E)**				
**ν_s_ PO_2_^−^**				
	**Mean rank difference**	**Signifficance**	***p*** **-value**	**Z score**
**GZ vs. SGZ**	−50.5	ns	0.063	2.56
**GZ vs. CA1**	−24.9	ns	>0.999	1.36
**GZ vs. CA3**	−109	***	<0.001	5.68
**SGZ vs. CA1**	25.6	ns	>0.999	1.27
**SGZ vs. CA3**	−58.8	*	0.031	2.8
**CA1 vs. CA3**	−84.4	***	<0.001	4.29
**(F)**				
**ν** **_s _** **PO_2_^−^ POSITION**				
	**Mean rank difference**	**Signifficance**	***p*** **-value**	**Z score**
**GZ vs. SGZ**	73.1	***	<0.001	5.06
**GZ vs. CA1**	92.8	***	<0.001	6.83
**GZ vs. CA3**	80.4	***	<0.001	5.38
**SGZ vs. CA1**	19.8	ns	0.959	1.41
**SGZ vs. CA3**	7.32	ns	>0.999	0.475
**CA1 vs. CA3**	−12.5	ns	>0.999	0.853

## Data Availability

The raw data supporting the conclusions of this article will be made available by the authors upon request.

## Supplementary Materials

The following supporting information can be downloaded at: https://www.mdpi.com/article/10.3390/cells14060435/s1, Figure S1: PCA score plots of different hippocampal layers and the SGZ under different experimental conditions.; Table S1: Main differential meaningful bands of SGZ, GZ, CA1 and CA3 hippocampal layers observed in second derivative average spectra and their contribution to PCA; Table S2: Main differential meaningful bands of WS, WE, TS and TE groups observed in second derivative average spectra and their contribution to PCA analysis.

## Abbreviations

The following abbreviations are used in this manuscript:

DG	Dentate gyrus
FTIR	Fourier transform infrared
SR	Synchrotron radiation
SGZ	Subgranular zone
TnC	Tenascin C
EE	Enriched environment
SC	Standard condition
GZ	Granular zone
TnC KO	Tenascin-C-deficient
HCA	Hierarchical cluster analysis
PCA	Principal component analysis
PC	Principal component
LDA	Linear discriminant analysis
RF	Random forest
WS	Wild type mice housed in standard conditions
WE	Wild type mice house in enriched environment
TS	TnC-deficient mice housed in standard conditions
TE	TnC-deficient mice housed in enriched environment
