# Supplementary material for: SR-FTIR Biomolecular Characterization of the Hippocampus: The Role of Tenascin C in Adult Murine Neurogenesis in the Subgranular Zone
Source: Cells. 2025 Mar 14;14(6):435. doi: 10.3390/cells14060435 (PMC11941197; doi:10.3390/cells14060435)
Supplement: Supplementary file 1 [file cells-14-00435-s001.zip › cells-3506484-supplementary.pdf]

SUPPLEMENTARY INFORMATION

SR-FTIR biomolecular characterization of the hippocampus: the role of tenascin C in adult murine neurogenesis in the subgranular zone

Milena Tucić[1], Andrej Korenić[1], Vera Stamenković[2], Tanja Dučić[3], Pavle Andjus[1*]

[1]Institute of Physiology and Biochemistry "Jean Giaja", Faculty of Biology, University of Belgrade, 11000 Belgrade, Serbia; milena.tucic@bio.bg.ac.rs (M.K.); andrej.korenic@bio.bg.ac.rs (A.K.)
[2]Center for Integrative Brain Research, Seattle Children's Research Institute, Seattle, WA 98105, USA; vera.stamenkovic@seattlechildrens.org
[3]ALBA-CELLS Synchrotron, 08290 Cerdanyola del Vallès, Spain; tducic@cells.es
*Correspondence: pandjus@bio.bg.ac.rs

This document includes:

Tables S1, S2

Figure S1

Table S1. Main differential meaningful bands of SGZ, GZ, CA1 and CA3 hippocampal layers observed in second derivative average spectra and their contribution to PCA. Related to Figure 4. ν - bond stretch; s – symmetric vibration; as – asymmetric vibration.

**Area of second derivative average spectra**	**Main differential meaningful bands (cm^{-1})**	**Main individual absorbances contributing to PCA**	**Main individual absorbances contributing to PCA**	**Main molecular correspondence**	**Reference**
Lipid (3100-2800 cm^{-1})		**PC1**	**PC2**		
	**3013**	3012	3017	νC=C	[30,41]
	**2960**	2957	2952	$\nu_{as}CH_3$	[11,41]
	**2922**	2921	2916	$\nu_{as}CH_2$	[11,41]
	**2872**	2879	2875	$\nu_{s}CH_3$	[41]
	**2852**	2852	2846	$\nu_{s}CH_2$	[9,41]
Protein and ester (1800-1480 cm^{-1})		**PC2**	**PC4**		
	**1742**	1742	1747	νC=O	[9,30]
	**1683**	1685	1689	β-turn (Amide I)	[30,41]
	**1656**	1658	1659	α-helix (Amide I)	[30,41]
	**1637**	1639	1640	β-sheet (Amide I)	[30,41]
	**1547**	1545		α-helix (Amide II)	[41]
	**1516**	1517	1514	Tyr residue	[54]
Nucleic acid and carbohydrate (1270-800cm^{-1})		**PC1**	**PC3**		
	**1239**	1230	1230	$\nu_{as}PO^{2-}$	[30]
	**1172**		1173	ν_{as}CO-O-C	[6]
	**1124**	1122	1121	ν_{s}RNA	[6]
	**1087**	1087		$\nu_{s}PO^{2-}$	[30]
	**1048**	1050	1050	$\nu_{s}PO^{2-}$	[6]

	**970**	964		C-O deoxyribose,<br>C-C DNA	[31]

Table S2. Main differential meaningful bands of WS, WE, TS and TE groups observed in second derivative average spectra and their contribution to PCA analysis. Related to Figure 7. ν - bond stretch; s – symmetric vibration; as – asymmetric vibration.

**Area of second derivative average spectra**	**Main differential meaningful bands (cm^{-1})**	**Main individual absorbances contributing to PCA**	**Main individual absorbances contributing to PCA**	**Main molecular correspondence**	**Reference**
Lipid (3100-2800 cm^{-1})		**PC1**	**PC2**		
	**3013**	3012	3018	$\nu C{=}C$	[30,41]
	**2960**	2958	2952	$\nu_{as}CH_3$	[11,41]
	**2922**	2922	2915	$\nu_{as}CH_2$	[11,41]
	**2873**	2869	2864	$\nu_{s}CH_3$	[41]
	**2852**	2852	2846	$\nu_{s}CH_2$	[9,41]
Protein and ester (1800-1480 cm^{-1})		**PC4**	**PC5**		
	**1741**	1738	1741	$\nu C{=}O$	[9,30]
	**1686**	1689		β-turn (Amide I)	[30,41]
	**1657**	1661	1655	α-helix (Amide I)	[30,41]
	**1639**	1640	1636	β-sheet (Amide I)	[30,41]
	**1546**	1543	1549	α-helix (Amide II)	[41]
	**1516**	1513	1515	Tyr residue	[54]
Nucleic acids and carbohydrate (1270-800 cm^{-1})		**PC1**	**PC2**		
	**1240**	1240	1232	$\nu_{as}PO^{2-}$	[30]
	**1167**	1173		ν_{as}CO-O-C	[6]
	**1087**	1089		$\nu_{s}PO^{2-}$	[30]
	**1052**	1054		$\nu_{s}PO^{2-}$	[6]
	**968**	963	965	C-O deoxyribose,<br>C-C DNA	[31]

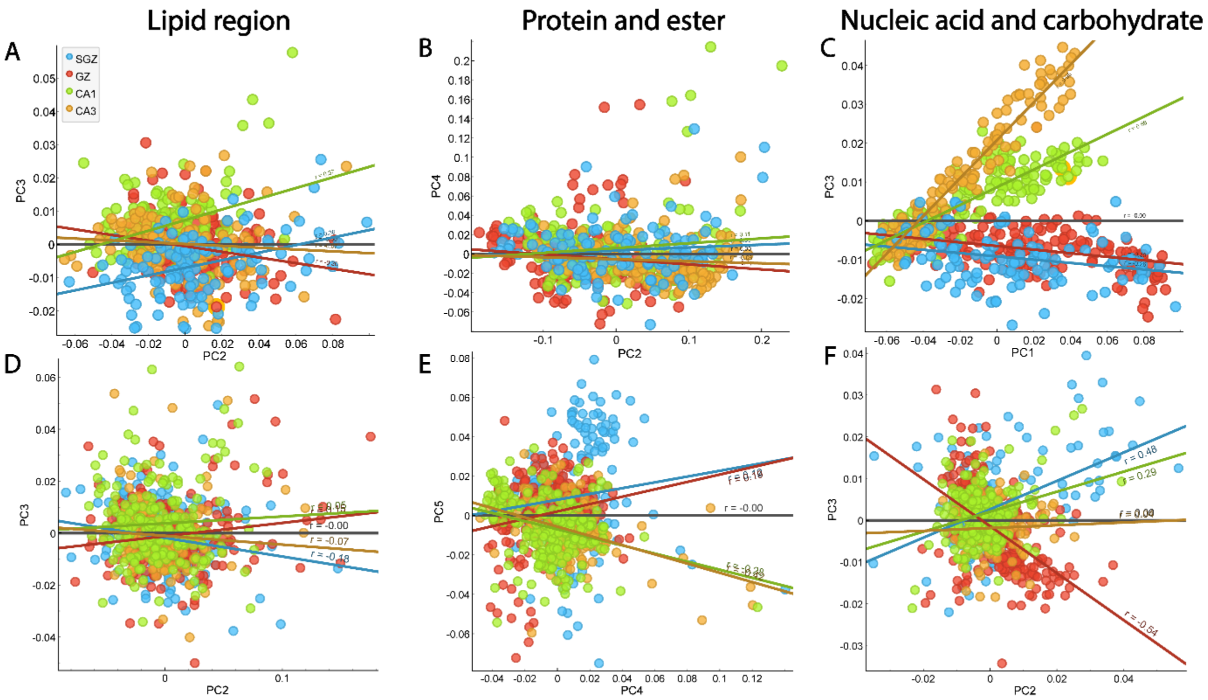

Figure S1: PCA score plots of different hippocampal layers and the SGZ under different experimental conditions. PCA and values of the PC scores for each region assigned above for the second derivative of the FTIR averaged spectra of different hippocampal layers (SGZ, GZ, CA1 and CA3) (A, B, C) and different experimental groups (WS, WE, TS and TE groups) (D, E, F). Graphs show the contribution of individual absorbance to the PCAs (loading values) of the specified principal components in blue and red. Related to Figs. 4 and 7.
